# Editorial: Biological Drivers of Vector–Pathogen Interactions

**DOI:** 10.3389/fcimb.2020.609495

**Published:** 2020-10-22

**Authors:** Ryan O. M. Rego, Job E. Lopez, Alejandro Cabezas-Cruz

**Affiliations:** ^1^Biology Centre, Institute of Parasitology, Czech Academy of Sciences, České Budějovice, Czechia; ^2^Faculty of Science, University of South Bohemia, České Budějovice, Czechia; ^3^Department of Molecular Virology and Microbiology, Baylor College of Medicine, Houston, TX, United States; ^4^Department of Pediatrics, National School of Tropical Medicine, Baylor College of Medicine, Houston, TX, United States; ^5^UMR BIPAR, INRAE, ANSES, Ecole Nationale Vétérinaire d’Alfort, Université Paris-Est, Maisons-Alfort, France

**Keywords:** vector, pathogen, immunity, transcriptome, arthropod, microbiome

## 

Blood feeding arthropods are a highly diverse group of animals that use blood as the main nutrient source. During this process, they transmit various viral, bacterial, and protozoal pathogens that are responsible for some of the worlds’ deadliest diseases leading to millions of human deaths as well as that of livestock every year. Understanding the relationships between vectors, pathogens, and the vector microbiota is an area of research, that used to be overlooked and now takes center stage when fighting vector borne diseases. The interactions include those seen at the time of acquisition, dissemination, and persistence of the pathogen within the vector and in the transmission to the vertebrate host. This basic research would help guide investigations into effective methods that would help block pathogen transmission (Shaw and Catteruccia, 2019). We believe the ‘biological drivers’ highlighted below are important in helping researchers develop tools that would alleviate the disease burden associated with vector-borne pathogens.

## Vector Microbiomes

Vector microbiomes drive key factors of the invertebrate host physiology, development, nutrition, vector competence, and pathogenesis. Since most of the information known regarding pathogen–microbiome interaction in mosquitoes has been conducted in laboratory reared insects, the work by Rodriguez-Ruano et al. compared mosquito sampling methods on microbiome profiles of wild caught mosquitoes. Furthermore, the research team compared the impact of insect preservation methods and pooled *versus* individual samples on the microbiome diversity. The work indicated that microbiome analysis of individual tissues produced little variation compared to whole specimens. Collectively, the study signified the importance of utilizing standardized methods for sample preservation, processing to minimize sampling bias.

Most microbiome studies in ticks are restricted to the taxonomic analysis of the tick microbiota with little insight about the functional roles of the non-pathogenic microbes associated with ticks. Obregón et al. analyzed the gut microbiome metagenomes of three tick species within the family Ixodidae. The results suggest that the tick microbiome forms a complex metabolic network that may increase microbial community resilience and adaptability. For example, the genes for biosynthesis of vitamin B, essential for tick survival, were present in the genomes of several gut microbiota bacteria. The presence of the same genes and functions in different bacteria of the microbiota implies functional redundancy. Hosting highly diverse and redundant microbiomes may offer ticks a competitive advantage in the environment. Guizzo et al. complement and expand these results by showing that the diversity of tick midgut microbiota is variable. Remarkably, these authors showed that tick midgut microbiota has a very low abundance compared with ovaries. This is a new take on tick microbiota that should be further explored.

## Vector Symbionts

Stewart and Bloom made an ambitious collection of literature about *Ixodes scapularis* symbionts and microbiome and their microbial interactions with the tick. The authors identified areas where research and conceptual development is needed. Particularly, a distinction between microbes adapted to using *I. scapularis* as a host or vector of transmission, and those that only circumstantially and transiently colonize the tick, is needed. The authors proposed that identifying the microbes that establish a long-term relationship with the tick might provide new insights into tick biology and tick–pathogen interactions. Other generalist microbes, widespread in the environment, may have pleiotropic and non-specific effects on the ticks.

## Vector Immunity

Argasid ticks are an understudied vector of emerging pathogens including African swine fever virus and relapsing fever spirochetes. Research on immunity in the New World argasid tick *Ornithodoros turicata* led to the identification of four defensin molecules which shared homology to defensins from the Old World tick *Ornithodoros moubata* (Armstrong et al.). The findings indicated that subsets of defensin molecules are produced after blood feeding while others are up-regulated in flat molted ticks. This work sets the foundation to now determine the role of defensins after pathogen acquisition.

## Vector–Pathogen Interplay

Most of the studies on tick**–**pathogen interactions focus on tick-borne bacteria (de la Fuente et al., 2017; Cabezas-Cruz et al., 2019). In this Research Topic, Hart et al. provide evidence that tick-borne encephalitis virus (TBEV) modifies gene expression in the salivary glands of infected *Ixodes ricinus*. Infected ticks were found to differentially express a number of genes coding for proteins involved in tick**–**host interactions. The genes include proteases, Kunitz-type serine protease inhibitors, cytotoxins, and lipocalins. Tick saliva components play an essential role in the initial virus transmission during tick feeding. The results suggest that sialome modulation may facilitate virus transmission during the early stages of tick feeding. Going more into the details of molecular mechanisms involved in tick**–**virus interactions, a different study provided evidence that tick-borne Langat virus (LGTV), related to TBEV, reduces the expression of a tick sphingomyelinase D (*Is*SMase), an enzyme that catalyzes the hydrolytic cleavage of lipids such as sphingomyelin (Regmi et al.). The authors further showed that *I. scapularis* ticks decrease *Is*SMase expression both *in vivo* and *in vitro*, which in turn produced an accumulation of sphingomyelin that supported membrane-associated viral replication and exosome biogenesis in tick cells.

Acinetobacter species have been identified in several insect species including human body lice. However, whether lice can acquire *A. baumannii* from the skin or blood of infected individuals and are able to transmit the bacteria to an uninfected host remain a topic of research. In their study, Ly et al. found a strong association between body lice infestation and the presence of *A. baumannii* DNA in the skin of homeless individuals from Marseille, France. The bacterium was not present in the blood of skin positive and/or lice infested individuals. The results of this study suggest that lice acquire *A. baumannii* while biting the skin of colonized individuals and likely transmit the bacteria in their feces.

Mixed infections within a vector can lead to competitive interactions and change the dynamics of how the pathogenic strains are transmitted or abundant within an ecological area. Dolores et al. showed that when strains of *Borrelia afzelii* were co-infecting the hard tick *I. ricinus*, it led to the reduction in spirochete load for both strains and seasonal treatment of the ticks did not lead to any effect on the intensity of inter-strain competition.

In order to understand how various pathogens are able to establish infection within their mammalian or arthropod host, it is important to determine what factors help them avoid the immune systems. The review by Lin et al. condenses current data studying the roles of various Borrelia associated Complement Regulator Acquiring Surface Proteins (CRASPs) and the importance of these molecules in tick-borne transmission and dissemination of the spirochete in mammalian hosts.

## Complexities of a Vector

An extremely well timed and extensive review on human head and body lice by Amanzougaghene et al. covers a host of sections connected with phylogenetics, epidemiology, disease**–**vector interactions as well as insecticide resistance. It certainly is a great resource collection of the current data that is available on one of the world’s oldest human parasites. It certainly will help researchers push forward with research that is lacking when it comes to understanding the human louse and its vectorial nature.

Finally, we would like to show our appreciation to all authors who contributed to the Research Topic and have opened more doors in the study of vector**–**pathogen interactions by providing major insights into these complex relationships.

## Author Contributions

All authors listed have made a substantial, direct and intellectual contribution to the work, and approved it for publication.

## Conflict of Interest

The authors declare that the research was conducted in the absence of any commercial or financial relationships that could be construed as a potential conflict of interest.
